# The parasitic worm product ES-62 protects against collagen-induced arthritis by resetting the gut-bone marrow axis in a microbiome-dependent manner

**DOI:** 10.3389/fitd.2023.1334705

**Published:** 2024-01-31

**Authors:** Margaret M. Harnett, James Doonan, Anuradha Tarafdar, Miguel A. Pineda, Josephine Duncombe-Moore, Geraldine Buitrago, Piaopiao Pan, Paul A. Hoskisson, Colin Selman, William Harnett

**Affiliations:** 1 School of Infection and Immunity, University of Glasgow, Glasgow, United Kingdom; 2 Strathclyde Institute of Pharmacy and Biomedical Sciences, University of Strathclyde, Glasgow, United Kingdom; 3 School of Biodiversity, One Health and Veterinary Medicine, University of Glasgow, Glasgow, United Kingdom

**Keywords:** arthritis, ES-62, gut, haematopoiesis, helminth, inflammation, osteoimmunology

## Abstract

The parasitic worm-derived immunomodulator, ES-62 rescues defective levels of IL-10-producing regulatory B cells (Bregs) and suppresses chronic Th1/Th17-driven inflammation to protect against joint destruction in the mouse collagen-induced arthritis (CIA) model of rheumatoid arthritis. Such autoimmune arthritis is also associated with dysbiosis of the gut microbiota and disruption of intestinal barrier integrity. We recently further exploited the CIA model to show that ES-62’s prevention of joint destruction is associated with protection of intestinal barrier integrity and normalization of the gut microbiota, thereby suppressing the gut pathology that precedes the onset of autoimmunity and joint damage in CIA-mice. As the status of the gut microbiota impacts on immune responses by influencing haematopoiesis, we have therefore investigated whether ES-62 harnesses the homeostatic mechanisms regulating this gut-bone marrow (BM) axis to resolve the chronic inflammation promoting autoimmunity and joint destruction in CIA. Reflecting this, ES-62 was found to counteract the BM myeloid/lymphoid bias typically associated with chronic inflammation and infection. This was achieved primarily by ES-62 acting to maintain the levels of lymphoid lineages (B220^+^ and CD3^+^ cells) observed in naïve, healthy mice but lost from the BM of CIA-mice. Moreover, ES-62’s ability to prevent bone-destroying osteoclastogenesis was found to be associated with its suppression of CIA-induced upregulation of osteoclast progenitors (OCPs) in the BM. Critically, and supporting ES-62’s targeting of the gut-BM axis, this rewiring of inflammatory haematopoiesis was lost in mice with a depleted microbiome. Underlining the importance of ES-62’s actions in restoring steady-state haematopoiesis, the BM levels of B and T lymphoid cells were shown to be inversely correlated, whilst the levels of OCPs positively correlated, with the severity of joint damage in CIA-mice.

## 1 Introduction

The Hygiene Hypothesis proposes that the rapid eradication of pathogens, which have co-evolved with humans over millenia, has left us with dysregulated and hyperactive immune systems. This generates chronic inflammation that has undoubtedly contributed to recent dramatic increases in allergic and autoimmune conditions, as well as ageing-associated comorbidities such as obesity, diabetes, cardiovascular disease and cancer (1–4). Reflecting this, human epidemiological data and animal model studies suggest that parasitic helminth (worm) infection might protect humans from developing such conditions and indeed, live helminths and/or their derived immunomodulatory products/mimetics have been trialed for efficacy in a range of inflammatory disorders (1, 2, 4, 5). One such product, ES-62, a phosphorylcholine (PC)-containing glycoprotein secreted by the filarial nematode, *Acanthocheilonema viteae*, protects against allergic, autoimmune and ageing-associated pathology in mouse models by downregulating aberrant TLR-MyD88 signalling to restore immunoregulation and thus homeostatically resolve chronic inflammation (4–6).

Although the precise detail of the immunoregulatory network remains to be defined, we have recently shown that the protection afforded by ES-62 against development of collagen-induced arthritis (CIA), a mouse model of rheumatoid arthritis, is associated with regularization of the gut microbiota. This is evidenced by normalization of Bacteroidetes : Firmicutes ratios, suppression of outgrowth of proteobacteria, and maintenance of bacterial diversity, particularly with respect to the butyrate-producing Clostridiales order and in addition, maintenance of intestinal barrier integrity, the disruption of which precedes autoimmunity and joint pathology in this model (7). Indeed, depletion of the gut microbiota, by use of a cocktail of broad-spectrum antibiotics (ABX), both greatly suppresses the induction of CIA and amelioration of any residual pathology by ES-62. Specifically, the ES-62-driven protection against each of CIA-induced intestinal barrier pathology and inflammation, autoimmunity (e.g., its reduction of pathogenic anti-type II collagen IgG2a antibodies, serum IL-6 levels and restoration of IL-10-producing Breg cells), osteoclastogenesis and joint pathology, can be seen to require an intact gut microbiome (7).

The ES-62-mediated promotion of increased health- and life-span in a high calorie diet (HCD) mouse model of obesity-accelerated ageing is similarly associated with the maintenance of gut health in terms of microbiota composition and barrier function (8). Interestingly, given that gut health impacts on the “training” of immune responses in the bone marrow (BM) and periphery (9–12), whilst HCD-induced gut dysbiosis and resulting inflamm-ageing was associated with accelerated ageing of haematopoietic stem cells (HSC) and consequent bias towards myeloid lineages, ES-62 acted to delay and reduce this dysregulation when administered to HCD-fed mice (13). HCD-induced dysregulation has been reported to be a result of HSCs responding both directly to microbiota-derived molecules and indirectly, via the impairment of the haematopoiesis-supporting BM microenvironment (11, 14, 15). As the primary target of ES-62, TLR/MyD88 signalling, plays key roles in these processes (9, 16, 17), we have investigated whether the ability of the parasitic worm product to harness the gut microbiome to facilitate its protection against CIA extends to targeting the gut microbiome-BM axis to “reset” haematopoiesis and consequently, maintain gut integrity and resolve the chronic inflammation driving inflammatory arthritis. We now show that ES-62 indeed targets the microbiome-BM axis to suppress the myeloid/lymphoid bias observed in the BM of CIA-mice. Its major action appears to be to maintain the levels of B220^+^ and CD3^+^ cells in the BM of CIA-mice with an intact microbiome and reflecting the importance of this, the levels of such B and T cells were found to negatively correlate with the degree of arthritis in mice. In addition, ES-62 suppressed the BM levels of the monocyte progenitors of osteoclasts, the cells involved in bone resorption during joint destruction in CIA.

## 2 Methods

### 2.1 Ethics statement

All procedures were performed in accordance with UK Home Office Project (PPL P8C60C865) and personal (PIL I518666F7, PIL 1675F0C46 and PIL ICEBDB864) licenses, following the “principles of laboratory animal care” (NIH Publication No. 86-23, revised 1985) and approval by the University of Glasgow Animal Welfare and Ethical Review Board.

### 2.2 Collagen-induced arthritis in mice

Male DBA/1 mice (Envigo, UK) were housed (in groups of 3 or 4, randomly allocated on arrival at 6-8 weeks of age) in the Central Research Facility (University of Glasgow, UK) and maintained, under specific pathogen-free conditions, at 22°C under a 12-h light/dark cycle with *ad libitum* access to water and Chow diet from Special Diet Services, UK as described previously (7). CIA was induced by intradermal injection of bovine Collagen Type II (CII -100 μg/dose; emulsified with complete Freund’s adjuvant; MD Biosciences) on day 0, followed by intraperitoneal challenge with 200 μg CII in PBS on day 21. Animals were treated with PBS or purified endotoxin-free ES-62 (2 µg/injection) subcutaneously on days -2, 0 and 21 and articular score of joint pathology determined as described previously (18–20). To deplete the gut microbiome, animals were given access to drinking water containing a cocktail of broad spectrum antibiotics (500 mg/L Vancomycin, 1g/L Neomycin and 1g/L Metronidazole) to eliminate Gram-positive, Gram negative and anaerobic microorganisms (21) 7 days prior to the induction of CIA and thereafter continuously throughout the experiment as described previously (7). Endotoxin-free ES-62 was purified from spent culture medium of adult *A. viteae* as described previously (18). Briefly, ES-62 was prepared from ~500 ml spent culture medium (endotoxin-free RPMI 1640 containing endotoxin-free glutamine (2 mM), endotoxin-free penicillin (100 U/ml), and endotoxin-free streptomycin (100 μg/ml)) of adult *A. viteae*. In order to remove larval forms (microfilariae) released by female worms the medium was passed through a 0.22-μm filter (Sigma-Aldrich, Poole, U.K.). It was then transferred to a stirred cell filtration unit containing a YM10 membrane (Amicon, Stonehouse, UK). After reducing the volume of the sample to 5-10 ml and transfer to endotoxin-free PBS, pH 7.2 (Cambrex Bioscience, Berkshire, U.K.), it was further concentrated to 200-500 μl using a Centricon microconcentrator with a 100-kDa cutoff (ES-62 exists in its native form as a tetramer with a molecular weight of ~240kD) membrane (Amicon). Purity (routinely, approaching 100% due to the apparent absence of other high molecular weight molecules (>100kD) in *A. viteae* E-S) and protein identity were confirmed by a combination of SDS-PAGE and Western blotting, using a rabbit antiserum specific for ES-62. Finally, the level of endotoxin in the ES-62 sample was confirmed using an Endosafe kit (Charles River Laboratories, Kent, U.K.). ES-62 is used at a working concentration that has an endotoxin reading of <0.003 endotoxin units/ml.

### 2.3 Flow cytometry

Flow cytometric analysis was performed as described previously (7, 13, 22). Briefly, following red cell-lysis (with 0.8% NH_4_Cl buffer), bone marrow (BM) cells were suspended in FACS buffer (PBS containing 2.5% BSA and 0.5 mM EDTA) and phenotyped using the following antibodies/fluorophores, with gating as described previously (7, 13, 22) and shown in Supplementary Figure 1.

**Table:** 

Specificity	Conjugate	Clone	Supplier	Catalogue #
Lin-	PE	145-2C11; RB6-8C5; RA3-6B2 Ter-119; M1/70	Biolegend	#133303
CD3	PE	17A2	BioLegend	#100205
B220	PE	RA36B2	BioLegend	#103207
Ter119	PE	TER-119	BioLegend	#116207
Sca-1	FITC	D7	BioLegend	#108116
Ly6C	PerCP- Cy5.5	HK1.4	BioLegend	#128011
Ly6G	APC	1A8	BioLegend	#127613
CD11b	APC	M1/70	BioLegend	#101212
CD11b	FITC	M1/70	BioLegend	#101206
CD45	APC	30-F11	BioLegend	#103112
CD115	Biotin	AFS98	eBioscience	#13-1152-82
CD117	APC	2B8	eBioscience	#17- 1171-82
CD117	Biotin	2B6	BioLegend	#105803
RANKL	Biotin	IK22/5	Biolegend	#510003
Streptavidin	APC-Cy7		BioLegend	#405208

LSK HSC analysis involved use of a PE-conjugated lineage cocktail, in combination with FITC anti-Sca-1 and APC or biotin anti-CD117 antibodies. Antibodies were employed at 0.2 µg/10^6^ cells (1/100 dilution) except for anti-CD45 (1/200 dilution). Streptavidin was used at 1/500 dilution. Cell death was assessed by fixed viability stain (APC-ef780) or 7AAD (BD Bioscience, UK) staining. Data were acquired using a FACS Canto flow cytometer and analysed using FlowJo Software (Tree Star In, OR USA, version 8.8.7) with populations being gated using isotype and fluorescence minus one (FMO) controls (7, 13, 22).

### 2.4 qRT-PCR

As described previously (7, 22), BM cells (10^6^) were lysed in RNeasy Lysis Buffer prior to mRNA extraction using RNeasy Plus Mini kit (Qiagen, Germany) and cDNA generated using the High Capacity cDNA Reverse Transcriptase kit (Applied Biosystems, Life Technology) and amplified using the StepOne Plus™ real-time PCR system (Applied Biosystems). KiCqStart® qPCR Ready Mix (Sigma-Aldrich) was utilised in conjunction with the following primer pairs:

**Table:** 

Gene	Forward Primer	Reverse Primer
RANKL, *tnfsf11*	TCTGTTCCTGTACTTTCGAG	TTCATGGAGTCT CAGGATTC
CXCL12, Cxcl12	GGAGGATAGATGTGCTCTGGAAC	AGTGAGGATGGA GACCGTGGTG
OPG, tnfrsf11b	GAAGATCATCCAAGACATTGAC	TCCTCCATAAAC TGAGTAGC
IL-1β, *illb*	GTGATATTCTCCATGAGCTTTG	TCTTCTTTGGGTA TTGCTTG
β-actin, *actb*	GATGTATG AAGGCTTTGGTC	TGTGCACTTTTA TTGGTCTC

Data were normalised to the reference gene β-actin to obtain the ΔCT values and the data expressed as Rq (2^-ΔCt).

### 2.5 Gut pathology

Ileum and colon tissues were fixed in either Carnoy’s solution (60% ethanol, 30% chloroform, and 10% glacial acetic acid), or 4% paraformaldehyde (PFA) prior to embedding in paraffin and storage at 4°C (23, 24). Although Periodic-acid Schiff (PAS)-staining intensity was considerably higher in the Carnoy’s fixed tissue, the pattern of responses was essentially identical. Prior to PAS staining, sections (7 µm) were dewaxed by washing in xylene, followed by hydration in decreasing concentrations of ethanol (100%; 95%; 70%), washing in deionised H_2_O and PAS staining using a standard protocol (7) and then mounted with Dibutylphthalate Polystyrene Xylene (DPX). Following PAS staining, digital images of blinded slides at 10x magnification were captured using an Olympus DP22 microscope digital camera attached to an Olympus BX43 microscope and three Fields of View (FoV) per section (triplicate sections per mouse) were imaged for intensity analyses by both semi-quantitative scoring using an ordinate scale between 1 and 6 (based on the overall intensity of pink staining) and Image J quantitative analysis of Mean Grey Values. Here, the digital images were converted to 8-bit greyscale such that every pixel is represented by a value between 0 (black; saturated) and 255 (white; no staining). Following subtraction of background and thresholding, the mean grey value of each section was subtracted from 255, the value for white, allowing for direct comparison with the ordinal semi-quantitative scoring system. Goblet cells were enumerated in 10 random FoVs per section with the data presented as mean values per mouse (triplicate sections) of average numbers derived from the FoVs.

For analysis of lectin binding, tissue sections (7 μm thickness) were dewaxed in xylene followed by hydration in ethanol (100%; 90%; 70%; 50%; 30%) before retrieving antigen in citrate buffer, pH 5.0 for 20 min at 95°C. Following treatment with Carbo-Free Blocking Solution (Vector labs) to minimise background staining due to glycoprotein contamination, sections were washed in PBS-T (PBS 0.05% Tween20) and blocking of endogenous biotin with streptavidin/biotin blocking solution was performed following manufacturer’s instructions (Vector laboratories, SP-2002). Sections were incubated with biotinylated Ulex europaeus (UEA)-1 lectin (1:200) to detect terminal alpha-L(1,2)-fucosylation and incubated overnight at 4°C. Following washing, cellular fucose expression was visualized by staining with streptavidin-Alexa 488 and counter-staining with Vectashield anti-fade mounting medium containing DAPI. Images were acquired using an EVOS microscope and analysed by Image J software.

### 2.6 Statistical analysis

Data were analysed using GraphPad Prism 10 software using unpaired student T-tests, Mann-Whitney tests, one or two-way ANOVA with Fishers LSD post-test for parametric data or Kruskal-Wallis test for non-parametric/ordinal data. The data presented in scatter plots are the mean values (of triplicate images/assays) or, for flow cytometric analysis, the % live cells or Mean Fluorescence Intensity (MFI) values of the individual mice in the group (bar is the median value for the group) and analysed by one-way ANOVA. For time-courses, the data are presented as the mean ± SEM values for the group (n values presented in legends) derived from the mean values for the individual mice and analysed by two-way ANOVA. Significant differences between the cohorts are shown on the figures, where significance is denoted by *p < 0.05, **p < 0.01, ***p < 0.001 and ****p<0.0001.

## 3 Results

### 3.1 CIA drives an inflammatory myeloid bias in the BM, particularly during the initiation phase prior to the onset of joint pathology

**Figure 1:**
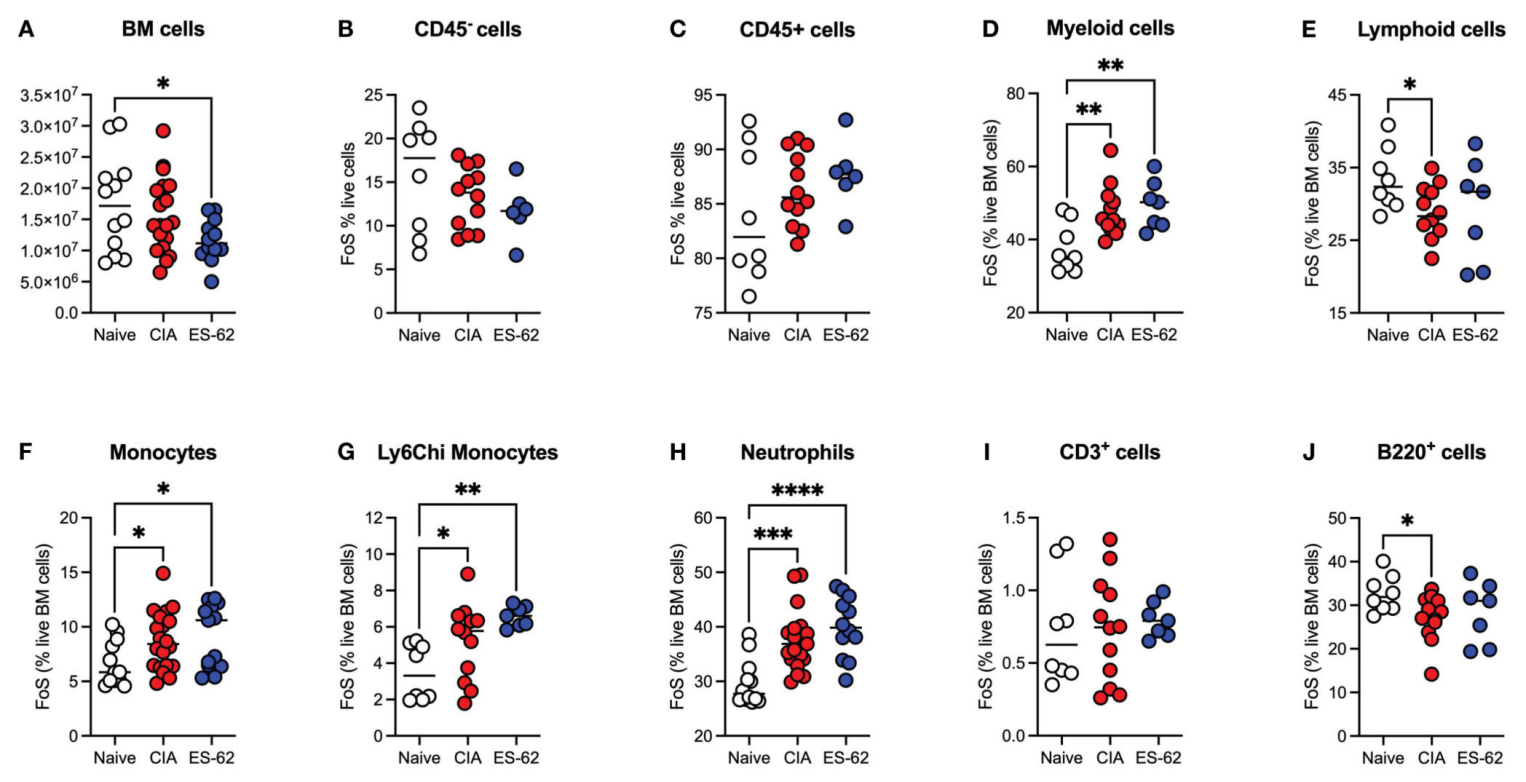
Modulation of BM lineages in CIA ± ES-62 mice. Data are presented as the values for individual mice (coloured symbols, bar represents median for group) in each cohort where significant differences are indicated by *p<0.05; **p<0.01, ***p<0.001 and ****p<0.0001 for numbers **(A)** and frequency of single cells (FoS) expressed as % of live BM cells (B-J) of total BM cells **(A)**, CD45^-^ **(B)**, CD45^+^ **(C)**, myeloid cells **(D)**, lymphoid cells **(E)**, monocytes **(F)** Ly6C^hi^ monocytes **(G)**, neutrophils **(H)**, T-lineage cells **(I)** and B-lineage cells (J). Data are pooled from 2-3 independent experiments and mice were culled when joint pathology was established (days 33-37) with articular scores: Naïve, 0; CIA-PBS, 4.16 ± 0.75 and CIA-ES-62, 2.42 ± 0.73 and where the CIA-PBS, but not the CIA-ES-62, group were significantly different (**p<0.01) from the Naïve mice.

Although there was a significant decrease in the total numbers of BM cells in the ES-62-treated CIA-(ES-62), relative to Naïve, mice, there were no significant differences between those in the Naïve and CIA (PBS-treated), or between the latter disease control and ES-62-treated CIA (ES-62) groups when the animals were culled in the established phase of joint disease (day 33-37; Figure 1A). Likewise, there were no significant differences in the proportions of CD45^-^ or CD45^+^ (Figures 1B, C) BM cells between the Naïve and CIA groups although there was a trend towards an increase in CD45^+^ and a decrease in CD45^-^ cells in the CIA (PBS or ES-62-treated) mouse groups, perhaps indicative of induction of CIA promoting production of haematopoietic lineages. However, whilst this increase in CD45^+^ cells was reflected in the proportions of myeloid cells in both cohorts of CIA-mice (Figure 1D), induction of inflammatory arthritis was accompanied by a significant decrease in the levels of lymphoid cells and treatment with ES-62 showed some evidence of normalising the proportions of these cells back to those observed in the BM of Naive mice (Figure 1E). The increase in myeloid cells was mirrored by increases in the proportions of each of total and inflammatory Ly6Chi monocytes and neutrophils in both the PBS- and ES-62-treated CIA mice (Figures 1F-H). By contrast, whilst there were no significant differences between the groups in terms of T (CD3^+^) lineage cells, the BM of CIA-PBS (CIA) but not CIA-ES-62 (ES-62) mice showed a decrease in the levels of B (B220^+^)-lineage cells, (Figures 1I, J).

**Figure 2:**
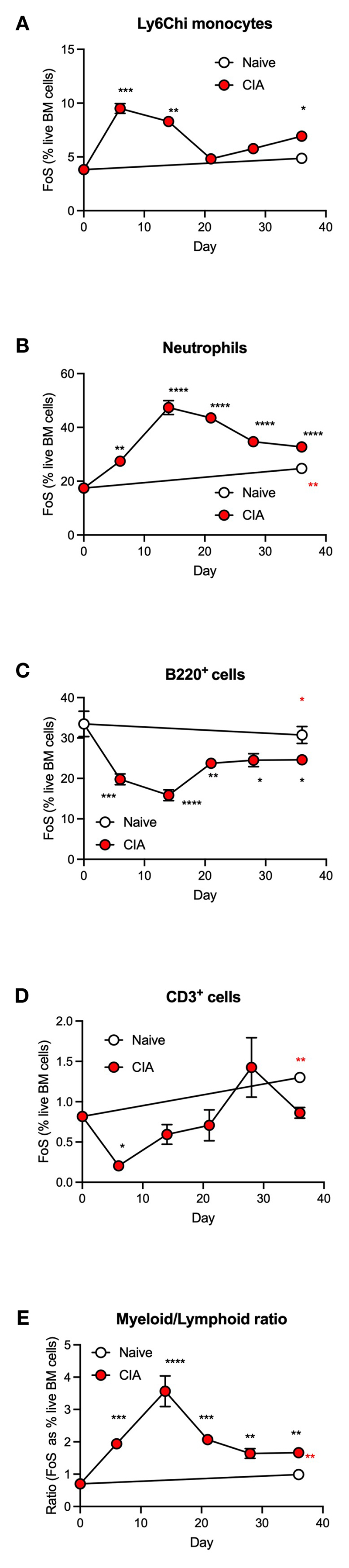
CIA is associated with an increased myeloid/lymphoid bias in the initiation phase of disease. Data are presented as the mean ± SEM values for 3 individual mice (coloured symbols) in naïve (days 0 and 36) and CIA-PBS groups where significant differences are indicated by *p<0.05; **p<0.01, ***p<0.001 and ****p<0.0001 (black* for CIA versus naïve at day 0 and red* for naïve versus CIA, both at day 36) for frequency of single cells (FoS) expressed as % of live of Ly6C^hi^ monocytes **(A)** neutrophils **(B)**, B-cells (**C**) and T-cells (**D**) over the phases of initiation (≤14 days), preclinical (day 21) and established joint pathology (≥ 28 days). The ratio of total myeloid/lymphoid cells (calculated on FoS values) throughout progression of the model is also presented (E). Data are from a single experiment.

Our previous studies established that gut damage and microbiome dysbiosis were pronounced by day 6 following primary immunization with type II collagen, preceding the development of autoimmunity, chronic inflammation and joint pathology (7). We therefore examined the kinetics of the CIA-induced dysregulation of haematopoiesis to determine whether this was associated with development of such gut pathology or the resultant chronic inflammation driving joint damage. This analysis showed the observed increase in Ly6C^hi^ monocytes and neutrophils (Figures 2A, B) and the decrease in B-lineage cells (Figure 2C) was most pronounced during the induction phase of CIA, with T-lineage cells also showing a significant decrease at this stage (Figure 2D). Such CIA-induced modulation of BM populations peaked within 6-14 days before returning towards the levels observed in naïve mice by day 21, but significantly altered proportions of all these populations were maintained during the established phase of chronic inflammatory arthritis. Overall, this disruption of haematopoiesis was manifested as a myeloid/lymphoid bias (Figure 2E), a state generally characterized as an inflammatory phenotype and one that likely contributes to the generation of autoimmunity. The secondary increase in monocytes observed following the booster CII immunization reflects the biphasic pattern of CIA-associated colon damage we reported previously (7) and may provide a rationale for disease flares in human RA.

### 3.2 ES-62 normalisation of the BM niche is dependent on an intact microbiome

**Figure 3:**
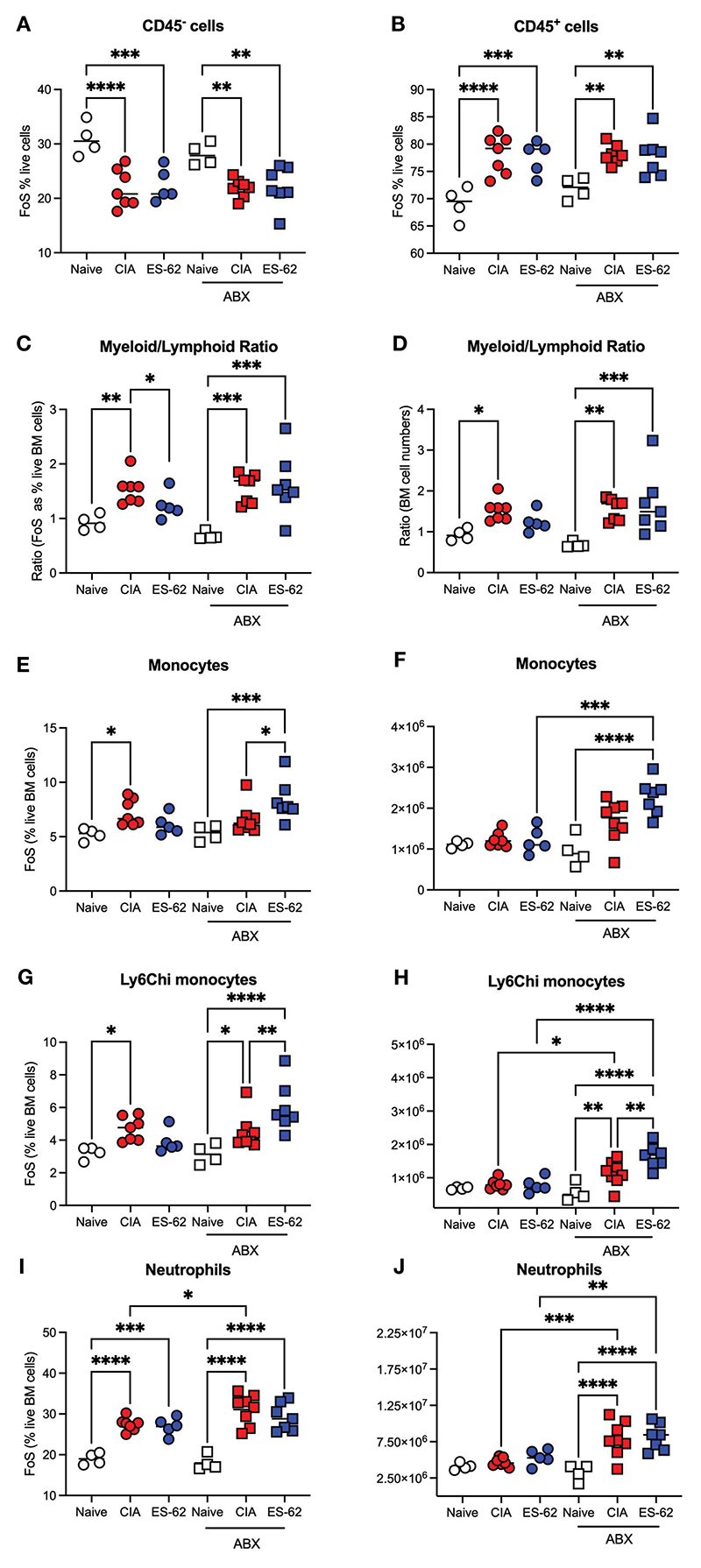
The impact of microbiome status on BM myeloid cell populations. Data are presented as the values for individual mice (coloured symbols, bar represents median of group) in each cohort and where microbiome depletion is denoted as “ABX” and square symbols. Significant differences are indicated by *p<0.05; **p<0.01, ***p<0.001 and ****p<0.0001 for FoS **(A, B, E, G, I)** or absolute numbers **(F, H, J)** for CD45^-^ cells **(A)**, CD45^+^ cells **(B)**, myeloid/lymphoid bias (C, D) calculated on the basis on FoS values and absolute numbers, respectively), monocytes **(E, F)**, Ly6C^hi^ monocytes (G, H) and neutrophils (I, J). Data are from a single experiment representative of 3 independent models and where (non-remitting) articular scores at cull (day 29) were Naïve, 0; CIA-PBS, 5.86 ± 1.12; CIA-ES-62, 0.4 ± 0.24 where ***p<0.001 for PBS versus Naïve and ES-62 and for the ABX-treated mice, Naïve, 0; CIA-PBS, 2.62 ± 0.99; CIA-ES-62, 4.86 ± 1.18 and where the latter two groups are not significantly different from each other but both are (*p<0.05 and **p<0.01, respectively) significantly different from their intact microbiome counterparts.

To further address the role of the microbiome dysbiosis and loss of gut barrier integrity in both CIA-associated disruption of haematopoiesis and its normalisation by ES-62, we investigated how these responses were affected by microbiome-depletion, resulting from providing the mice with broad spectrum antibiotics (ABX) in their drinking water from 7 days prior to and then throughout the CIA model (7). This approach showed that the proportions of CD45^-^ and CD45^+^ cells were again decreased or increased, respectively, in both cohorts of CIA-mice, irrespective of their microbiome status (Figures 3A, B). However, whilst exposure to ES-62 counteracted the increased myeloid/lymphoid bias (calculated either from the % live BM cells or absolute cell counts) in mice with an intact microbiome, this protection was lost in ABX-treated CIA-mice (Figures 3C, D) indicating the requirement for gut microbiota for such ES-62-protection. Again, the protective effect of ES-62 against this myeloid bias in CIA-mice did not reflect significant suppression of the CIA-driven rise in total or inflammatory Ly6C^hi^ BM monocytes (Figures 3E-H) or neutrophils (Figures 3I, J) and these increases in myeloid cells were not suppressed by microbiome depletion but rather, if anything, increased. Although in this experiment the proportions of these monocyte populations in the BM of CIA-ES-62 mice also tended to increase relative to Naïve animals, albeit this did not reach significance, overall there was a general lack of effect of ES-62 on the increased proportions and numbers of myeloid lineage cells in the BM of CIA-mice, culled during the established phase of joint pathology (Figures 1, 3). Also, although some of the changes in absolute numbers of these cells did not reach statistical significance, they showed a similar profile to the changes in the proportions of these populations (Figures 3E-H).

**Figure 4:**
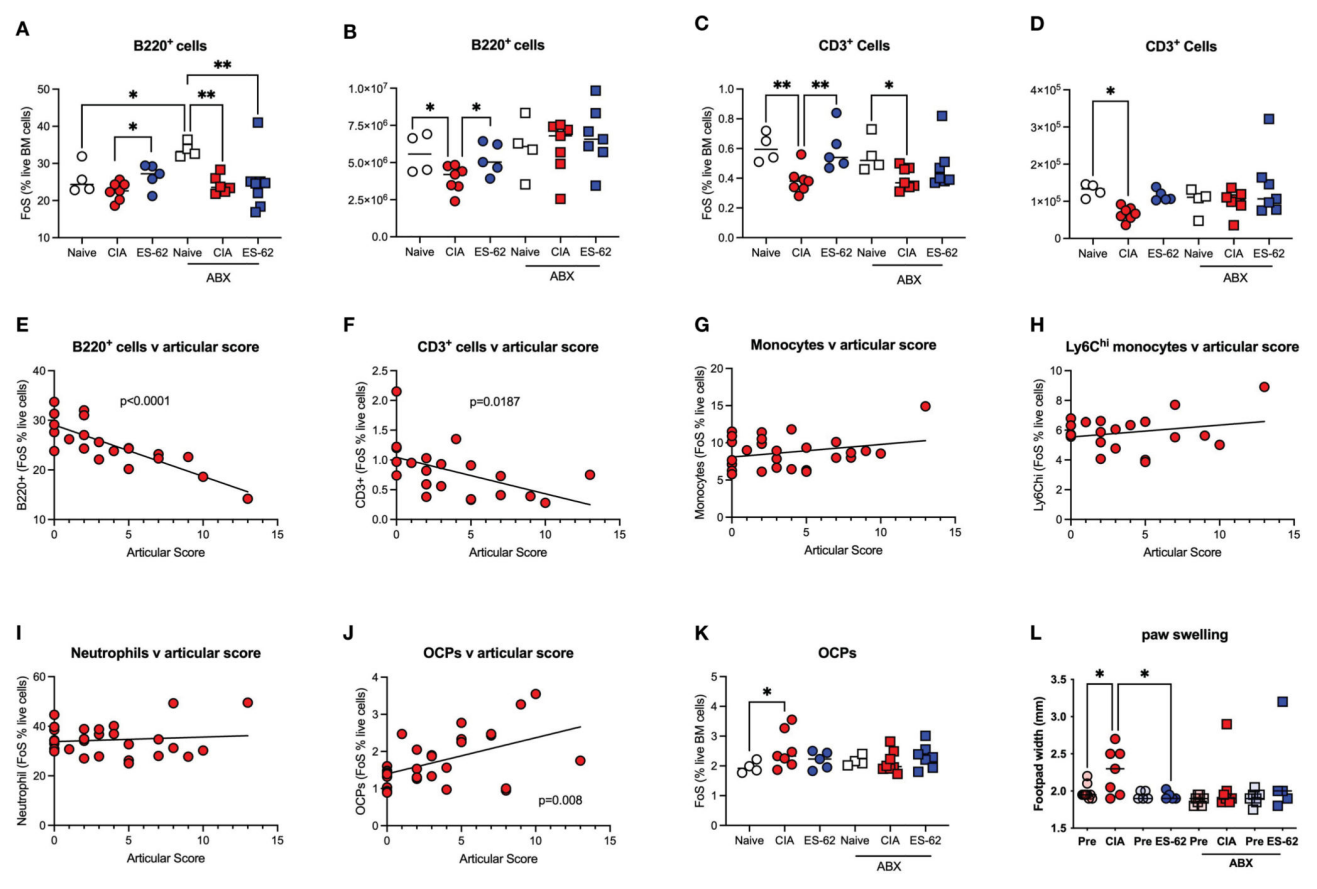
ES-62-protection of lymphoid lineages is microbiome-dependent and contributes to amelioration of joint pathology. Data are presented as the values for individual mice (coloured symbols, bar represents median of group) in each cohort and where microbiome depletion is denoted as “ABX” and square symbols. Significant differences are indicated by*p<0.05 and **p<0.01 for FoS **(A, C)** or absolute numbers **(B, D)** for B cells **(A, B)** and T cells **(C, D)**. Data are from the single experiment described in Figure 3 and are representative of 2 independent models. Linear regression analysis of levels (FoS; % live BM cells) of B lineage cells **(E)**, T lineage cells **(F)**, monocytes **(G)**, Ly6Chi monocytes **(H)**, neutrophils **(I)** and OCPs (**J**) versus articular scores was performed on the data from CIA-PBS mice from 3-4 independent experiments and where significant correlations are indicated by the annotated p values. The levels of OCPs (FoS; (K)) and joint pathology **(L)**, as evidenced by paw swelling at day 29 cull versus at day 21 prior to secondary CII immunization [relevant “pre” groups] are presented. Data **(K, L)** are from the single experiment described in Figure 3, and where significant differences are indicated by *p<0.05.

By contrast, ES-62 acted to limit the CIA-induced decline in proportions and numbers of BM B cells (Figures 4A, B) and T cells (Figures 4C, D) and this protection was lost in the corresponding microbiome-depleted cohort. Perhaps reflecting these protective actions of ES-62, whilst linear regression analysis of the mice in this study showed a clear inverse relationship between the levels of both B220^+^ and CD3^+^ cells in the BM of CIA mice and joint disease severity (Figures 4E, F), no significant correlation was found between articular score and any of the myeloid lineages tested (Figures 4G-I). However, and consistent with our previous studies that ES-62 suppresses development of joint disease (articular score and joint pathology) in CIA, at least in part by impacting on osteoclastogenesis (reduced osteoclast progenitors [OCPs], cathepsin K expression in joints, *ex vivo* functional maturation [TRAP+ staining of multinucleated OCs and bone resorption]), we now show that the levels of BM OCPs, a monocyte subset, correlate with joint damage in CIA (Figure 4J) and that microbiome depletion prevents the CIA-induced increase (Figure 4K). As the levels of OCPs in the BM of CIA-ES-62 mice are not significantly different from those in the Naïve mice, these data suggest that ES-62 acts to suppress the elevated levels of BM OCPs in CIA-PBS mice (Figure 4K) and are consistent with its ability to suppress joint damage (as evidenced by paw width; Figure 4L). Notably, treatment of naïve mice with ES-62 did not result in modulation of the myeloid/lymphoid ratio, or the levels of myeloid or lymphoid lineages *per se*, in the bone marrow of DBA/1 mice (results not shown).

### 3.3 Targets of ES-62 action in the BM and gut

**Figure 5:**
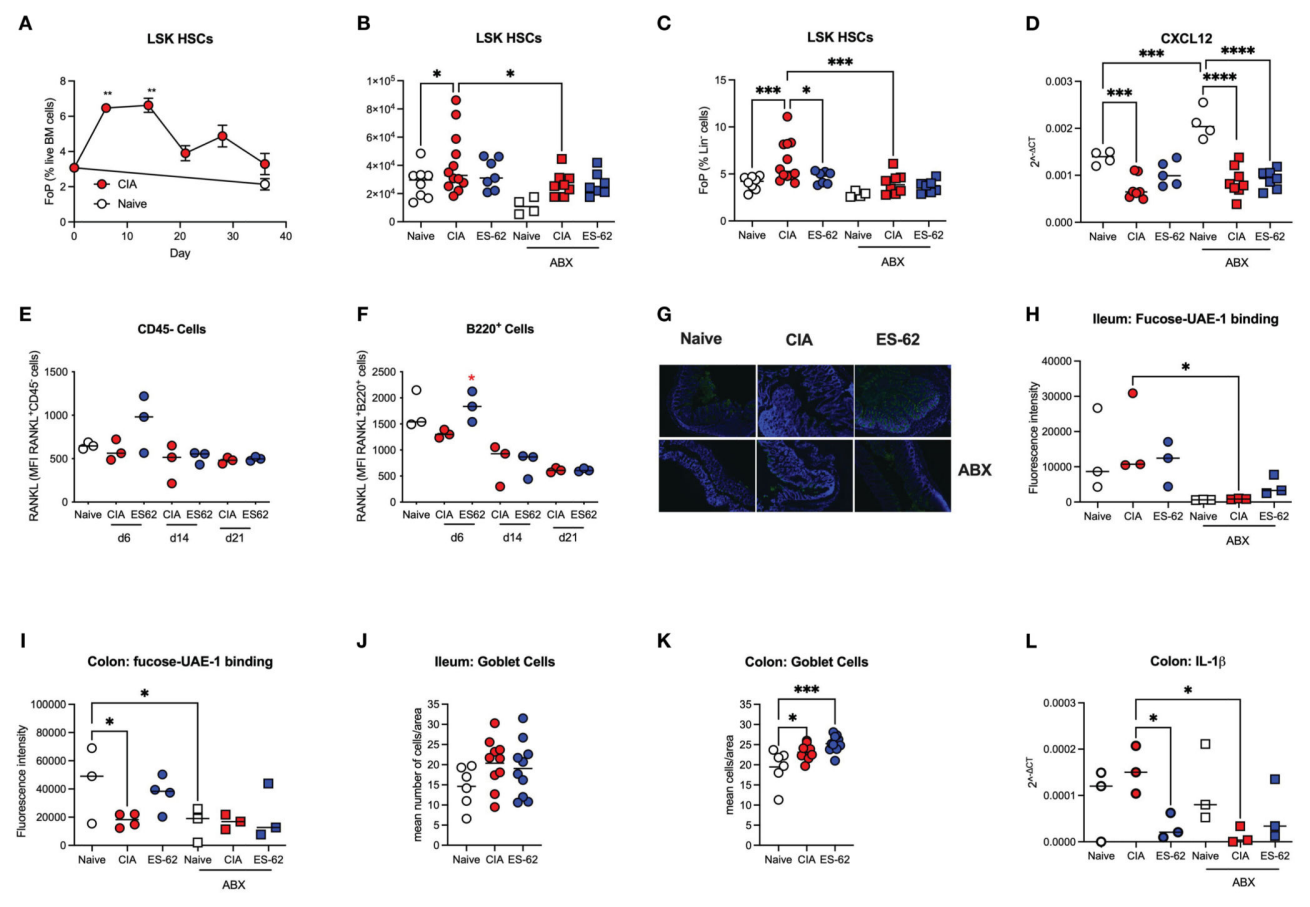
Targets of ES-62 in the BM and gut. **(A)** Data are presented as the mean ± SEM values for 3 individual mice (coloured symbols) in Naïve (days 0 and 36) and CIA-PBS groups (time points as indicated) where significant differences are indicated by **p<0.01 for the levels of Lin^-^Sca-1^+^c-kit^+^ (LSK) HSC (as % Lin^-^ cells [frequency of precursor (FoP)]) over the phases of initiation (≤14 days), preclinical (day 21) and established joint pathology (≥ 28 days). Data are from the single experiment described in Figure 2. **(B-D)** Data are presented as the values for individual mice (coloured symbols, bar represents median of group) in each cohort and where microbiome depletion is denoted as “ABX” and square symbols. Significant differences are indicated by *p<0.05, ***p<0.001 and ****p<0.0001 for Lin^-^Sca-1^+^c-kit^+^ (LSK) HSCs (as absolute numbers (**B**) or (**C**) % Lin^-^ cells [frequency of precursor (FoP)] and data are pooled from 2 experiments). BM CXCL12 mRNA levels are from the single experiment (of two) described in Figures 3, 4 **(D). (E, F)** Data are presented as the values of RANKL expression (MFI) for 3 individual mice (coloured symbols) RANKL^+^CD45^-^ (**E**) and RANKL^+^B lineage **(F)** cells. Significant differences are indicated by *p<0.05 for RANKL expression by B220^+^ cells in the BM between the CIA-PBS and CIA-ES-62 groups. Levels of terminal fucosylation (as evidenced by Image J imaging of intensity of UEA-1 staining, see representative images of colon tissue **[G]**) are presented for ileum and colon tissue **(G-I)**. Goblet cell numbers in ileum and colon tissue **(J, K)** were enumerated from PAS-stained sections and data are presented as the mean values for individual mice (coloured symbols, bar represents median of group). IL-1β mRNA expression in colon tissue (**L**) was determined by qRT-PCR and the data presented as the mean Rq values for individual mice (coloured symbols, bar represents median of group) in each cohort and where microbiome depletion is denoted as “ABX” and square symbols. Significant differences in panels **(H-L)** are indicated by *p<0.05 and ***p<0.001.

The mechanisms underlying the ES-62 protection of B and T cell levels in the BM are not clear, but inflammation impacts on HSC functionality, promoting both further inflammatory myeloid bias and HSC senescence. We therefore next investigated the effects of CIA on the levels of Lin^-^Sca-1^+^c-Kit^+^ (LSK) HSCs in the BM of such mice and this revealed a rapid transient increase in LSK HSCs during the initiation of CIA with levels remaining elevated throughout the established phase of joint pathology (Figures 5A-C). The higher numbers and proportions of LSK HSCs observed in CIA were counteracted by exposure of such mice to ES-62 and depletion of the microbiome (Figures 5B, C). In addition, the production of cytokines and growth factors like RANKL, OPG, IL-7 and CXCL12 by stromal cells and osteoblasts, as well as autocrine signalling by RANKL, have been reported to be important for B cell development (25, 26). Perhaps reflecting this, whilst there were no significant differences in the levels of BM OPG mRNA (results not shown), there was a significant reduction in CXCL12 mRNA levels observed in the BM from CIA-PBS mice, relative to the Naive animals. As the levels of CXCL12 mRNA found in the BM from CIA-ES-62 mice were not significantly different from those in the Naïve group, these data suggested that the CIA-PBS-associated decrease was partially reversed by ES-62 treatment. Moreover, such ES-62 rescue was abrogated by microbiome depletion (Figure 5D). However, there were no differences in the levels of cell surface expression of RANKL (or RANKL mRNA expression in whole BM extracts) on CD45^-^ or B lineage cells amongst any of the groups during the established phase of joint disease (results not shown). By contrast, there were elevated levels of cell surface RANKL expression (although not significant in the case of the CD45^-^ cells) in the CIA-ES-62 cohort during initiation (day 6) of CIA (Figures 5E, F and Supplementary Figure 2), albeit the accompanying elevation in the levels of B lineage cells observed at days 6, 14 and 21 in these mice did not reach significance (results not shown).

Terminal fucosylation of the intestinal epithelia and its regulation of the mucus layer and inflammation has been reported to promote intestinal barrier integrity and microbiome homeostasis (24, 27–29) and thus we investigated how these gut parameters were affected by CIA, ES-62 and microbiome status. This showed that whilst there were no significant differences in terminal fucosylation of the ileum (as evidenced by binding of UEA-1) amongst the naïve and CIA ± ES-62 groups, CIA was associated with a decrease in the (much higher) terminal expression of fucose found in the colon and this was rescued by ES-62 treatment (Figures 5G-I). These data also confirmed our previous findings that treatment with ES-62 protected against loss of colon structural integrity observed in CIA (7). Consistent with such fucosylation being maintained and regulated by the gut bacteria, the levels at both sites were significantly reduced by microbiome depletion (Figures 5G-I). Reduced colon fucosylation has been associated with goblet cell hyperplasia, inflammation and colitis and consistent with this, the numbers of goblet cells were increased in colon but not ileum sections (Figures 5J, K) of CIA-mice but this was not modulated by exposure to ES-62 and there were no differences in overall PAS staining of mucins amongst these groups (results not shown). By contrast, IL-1β mRNA levels were elevated in the colon of CIA-mice and suppressed by both ES-62 and microbiome depletion (Figure 5L).

## 4 Discussion

Induction of inflammatory arthritis in a variety of mouse models involves, at least in part, microbiota dysbiosis and disruption of intestinal barrier integrity (7, 21, 30–35). As far as we aware, we were the first to characterize the microbiome dysbiosis occurring in the ileum and colon and show that such dysbiosis and gut pathology occurs during the initial stages of disease initiation (as early as by day 6 in the CIA model), preceding the onset of autoimmunity and joint damage (7). Consistent with this central role of the gut, ABX-depletion of the microbiome suppresses the progression and severity of arthritis. Moreover, the ability of parasitic helminth-derived immunomodulator, ES-62 to reset immunoregulation (by restoring levels of IL-10-producing regulatory B cells [Bregs]) and exert its protection against Th1/Th17-driven autoimmunity and resultant joint pathology in the CIA model of RA, is lost following microbiome depletion (7). Reflecting this, ES-62’s protection against CIA is associated with its maintenance of intestinal barrier integrity and normalization of the gut microbiota, and even fostering gut-protective butyrate-producing Clostridiales genera implicated in maintaining inflammation homeostasis (5, 7). Thus, as it is well established that the gut microbiota and its products influence immune responses by modulating the BM microenvironment and cellular differentiation (9, 10, 36), we investigated whether ES-62 harnesses this gut-BM axis to rewire haematopoiesis and resolve the chronic inflammation fostering autoimmunity and joint destruction in CIA. Exposure to ES-62 was indeed found to impact on BM haematopoiesis in CIA-mice, acting to counteract the myeloid/lymphoid bias typically associated with the chronic inflammation that underpins autoimmune diseases and comorbidities associated with ageing, including inflammatory arthritis (11–14). ES-62 appears to do this primarily by protecting against the loss of lymphoid lineages (B220^+^ and CD3^+^ cells) otherwise observed in the BM of CIA-mice and, supporting its targeting of the gut-BM axis, this is only achieved in those animals with an intact microbiome. Underlining their importance in restraining progression to arthritis, the levels of B and T lymphoid cells pertaining in the BM were shown to be inversely correlated with severity of joint damage in CIA-mice.

In addition to its ability to suppress autoimmunity and resolve chronic inflammation in CIA, ES-62 also protects against joint disease by inhibiting CIA-promoted osteoclastogenesis and resultant bone resorption (7, 22). Reflecting the importance of dysregulation of haematopoiesis in contributing to all aspects of induction of inflammatory arthritis, our new data also show that CIA promotes significantly increased levels of the monocyte-derived OCPs, in a microbiome-dependent manner and the levels of these progenitors in the BM of CIA-mice correlate with severity of joint pathology. By contrast, the levels in the CIA-ES-62 group were not significantly different to those in Naïve mice, again supporting the proposal that such pathological “training” of BM progenitors is counteracted by exposure of CIA-mice to ES-62. Interestingly, there is no correlation between joint pathology and the levels of OCPs in the BM of CIA-ES-62 mice (results not shown), suggesting that ES-62 reduces both their numbers and their functionally “aggressive” phenotype. These data therefore complement our earlier findings that OCPs from CIA-mice exhibit greater *in vitro* osteoclastogenic potential than their counterparts from either Naïve or ES-62-treated CIA-mice (7, 22). That such increased osteoclastogenic potential was also dependent on an intact microbiome (7) suggests that the CIA-associated gut dysbiosis “trains” the BM environment to produce more “aggressive” OCPs, consequently driving bone destruction. Bone remodelling is normally tightly controlled by the counter-regulatory actions of osteoclasts (OCs; bone resorption) and osteoblasts (OBs; bone formation): thus, in addition to suppressing osteoclastogenesis, ES-62 may also promote functional OB maturation. Although not investigated directly, such activity may be indicated by the ability of ES-62 to maintain “normal” levels of B220^+^ cells, as B cell development in the BM requires the microenvironment and growth factor (e.g., CXCL12) production provided by the functional osteoblastic/stromal (CXCL12-abundant reticular [CAR] cells) niche (25, 26). Consistent with ES-62 maintaining this niche, we show here that ES-62 counters the reduction of CXCL2 observed in the BM of CIA-mice. Moreover, we have previously shown that ES-62 can act to protect this B cell-fostering niche by preventing the adipocyte/OB bias observed in high calorie diet (HCD)-fed male C57BL/6 mice, a model of obesity-accelerated ageing, also underpinned by gut microbiome dysbiosis, loss of intestinal barrier integrity and chronic inflammation (13). Furthermore, although no link with gut status was investigated, a recent study reported that E-S products from *Fasciola hepatica* [FHES; (37)] can rewire HSC and monocyte precursor differentiation towards an anti-inflammatory phenotype (Ly6C^low^ monocytes) and that transfer of such HSCs can ameliorate pathology in the experimental autoimmune encephalomyelitis mouse model of multiple sclerosis, a disease in which microbiome dysbiosis has been implicated as playing an important role (38). Collectively therefore, these data suggest that targeting the gut-BM axis may be a key mechanism by which parasitic worms exert immunoregulation and promote their survival and thus, that understanding these processes may signpost novel therapeutic interventions in chronic inflammatory disease.

Perhaps surprisingly, given its ability to suppress pathogenic effectors like IL-6, IL-17 and anti-CII IgG2a autoantibodies in a microbiome-dependent manner [results not shown for the present experiments and (7, 22)], the increased levels of monocytes and neutrophils observed in the BM of CIA-mice were not generally modulated by their exposure to ES-62. However, the (increased) levels of total and inflammatory Ly6^hi^ monocytes and neutrophils observed in the BM of CIA-mice were not found to correlate with the degree of pathology observed, nor were they abrogated by microbiome-depletion. Nevertheless, as the most profound disruption of steady-state haematopoiesis occurred during the initiation phase of CIA (≤ day 14), the period associated with the onset of gut pathology, this lack of effect of ES-62/microbiome-depletion may simply reflect that the elevated levels of these lineages pertaining during established arthritis are maintained by the chronic inflammation resulting from early changes in the gut microbiota and loss of intestinal barrier function, rather than by ongoing dysbiosis. Alternatively, although overall ES-62 does not significantly impact on the levels of myeloid cells, this may reflect its potential ability to retain in the BM, inflammatory myeloid cells otherwise recruited to the periphery and/or impact on their functional phenotype (e.g., by increasing the relative levels of myeloid derived suppressor cells). Indeed, our previous studies have shown that macrophages and dendritic cells derived from the BM of CIA-ES-62 mice, *in vitro*, exhibit dampened inflammatory responses relative to their CIA-PBS counterparts [reviewed in (4, 5)]. In any case, once again the normalisation of BM haematopoiesis in CIA-ES-62 mice is strikingly reminiscent of ES-62’s protection of gut health and resolution of chronic myeloid-driven inflammation in obesity-accelerated ageing, being similarly associated with normalization of the proinflammatory myeloid/lymphoid bias, again primarily by maintenance of lymphoid, particularly B lineage, cell levels in the BM of HCD-fed male C57BL/6 mice (13). Collectively, our data from these two diverse inflammatory disease models underline the central role that dysregulation of the gut-BM axis plays in driving autoimmunity and other ageing-associated comorbidities and support the unifying hypothesis that ES-62 harnesses normalisation of this axis to homeostatically reset haematopoiesis to a “steady-state”-like phenotype and hence, resolve chronic inflammation.

The mechanisms by which maintenance of BM lymphoid lineages contribute to the protective actions of ES-62 remain to be delineated but there is increasing evidence that mature populations of B220^+^ and CD3^+^ cells traffic to the BM and can impact on haematopoiesis (39–41). For example, some 30% of T cells in the BM (CD3^+^ cells typically constitute 1-5% of BM cells) are CD45Ra^+^CXCR4^+^Foxp3^hi^CD25^hi^ Tregs (39) recruited there by CXCL12, potentially to survive and mature and provide a reservoir of naïve Tregs in healthy individuals (40). These Tregs have been reported to be functionally more immunosuppressive than their systemic counterparts and may contribute to the perivascular HSC niche that promotes HSC quiescence and functional integrity (40). However, both helper and regulatory CD4^+^ T cells appear to have the capacity to control HSC and progenitor cell activity by modulating the production of myeloid differentiation-promoting cytokines, by regulating stromal cell function and IL-3-driven myelopoiesis, as well as adjusting the levels of IL-7 to promote lymphopoiesis and hence, maintain steady-state haematopoiesis (39, 40). Interestingly therefore, whilst we find the levels of BM CXCL12 mRNA to be reduced in CIA-mice, they are restored in ES-62-treated animals which have not been microbiome-depleted. However, the actions of ES-62, in a wide range of models of inflammatory disease including CIA (4, 5), do not reflect restoration of defective systemic Treg levels (7, 42) and indeed, ES-62 is ineffective in mouse models dependent on Tregs for protection against disease progression (7, 43). Nevertheless, many of the protective effects of mature lymphoid cells in the BM appear to involve IL-10 (39–41) and not only can ES-62 restore the levels of a range of IL-10-producing B cells in CIA-mice (7), at least some of these Bregs (T2-MZP phenotype) can convert CD4^+^ T cells to Tregs (41) which, rather than act systemically, could potentially home to the BM to protect HSC function. Alternatively, any protective effects of the restored levels of T cells in ES-62-treated CIA-mice could simply reflect the actions of helper T cells recruited to the BM (39, 40).

Reflecting emerging evidence for a role of B cells in regulating BM haematopoiesis, Bregs have been reported to be reduced in the BM of an ovariectomised mouse model of osteoporosis, a defect consistent with the loss of their ability to inhibit osteoclastogenesis (44). Interestingly in terms of the gut BM axis, administration of *Bifidobacterium longum* also suppressed osteoclastogenesis and the actions of this probiotic are associated with the generation of Bregs, efficient at suppressing osteoclastogenesis and dampening inflammation by modulating the Treg/Th17 balance in this model (45). Moreover, B cell-specific production of acetylcholine (ACh) has been shown to restrain both steady-state and emergency haematopoiesis, most likely by limiting HSC proliferation (46). Consistent with this proposal, conditional B-lineage (CD19^+^)-specific choline acetyltransferase (ChAT)-deficient mice exhibited increased levels of LSK HSCs and common myeloid, but not lymphoid, erythroid or platelet, progenitors. Moreover, these common myeloid progenitors exhibited greater proliferative potential, resulting in increased levels of monocytes and neutrophils, with more inflammatory phenotypes (46). Furthermore, the use of ACh receptor (Chrna7)-deficient mice indicated that CXCL12-BM stromal cell signalling played a role in restraining such increased myelopoiesis (46), reminiscent of the IL-1R/TLR-MyD88-driven “emergency” haematopoiesis triggered by inflammation and/or microbiome dysbiosis (16).

Notably for the hypothesis that B cells play a central role in maintaining steady-state haematopoiesis, B lymphopoiesis is itself compromised in autoimmunity and ageing (47) where chronic IL-1R/TLR4 signalling stimulates HSC proliferation and drives the proinflammatory myeloid/lymphoid bias (16, 48–50) by promoting (i) preferential development of myeloid cells from HSCs and dendritic cells from common lymphoid progenitors; (ii) induction of apoptosis of developing B cells and (iii) egress of developing B cells from the BM (48, 49). The aberrant escape of immature B cells from the BM is fostered by inflammation-driven downregulation of CXCL12 and is likely to perpetuate the vicious autoimmune inflammatory cycle by the further generation of autoreactive specificities (due to the less rigorous peripheral, relative to central, tolerance mechanisms) and resultant chronic inflammation feeding back on BM haematopoiesis (48, 49).

Thus, as TLR/MyD88 signalling is the primary target of ES-62 (4), our working model is that it subverts this proinflammatory signalling cassette both in the gut and BM to suppress inflammation and rewiring of HSCs towards “emergency” haematopoiesis, thereby rescuing lymphoid cell levels to further restore homeostasis. Supporting this, whilst suppressing BM MyD88 mRNA (7) and promoting CXCL12 mRNA and B cell levels, ES-62 reduces total BM cell numbers and counters the CIA-driven increase in the proportion and numbers of LSK HSCs in the BM of CIA-mice with an intact, but not depleted, microbiome. Pertinent to the generality of ES-62 in targeting the gut-BM axis to resolve chronic inflammation, it is also worth noting that B cell-specific depletion of MyD88 results in abrogation of pathogenic autoantibody responses (51–53) and is associated with the ES-62-induction of protective Bregs in mouse models of systemic lupus erythematosus (SLE), asthma and RA (7, 54, 55). Interestingly, the ability of ACh-producing B cells to regulate myeloid-driven inflammation may not be restricted to the BM as in addition to their homeostatic actions in the BM, these cells also appear to be involved in fine-tuning splenic haematopoiesis to further restrain cardiovascular inflammation following myocardial infarction (46). Likewise in the adeno-associated virus PCSK9 mouse model of atherosclerosis, whilst the dysregulation of BM haematopoiesis in B cell-ChAT-deficient mice was reflected by higher levels of CD11b^+^ myeloid cells, Ly6G^+^ neutrophils, Ly6C^hi^ monocytes and F4/80^+^ macrophages in the aorta and increased lesion size, relative to their control counterparts, the authors also found recruitment of ChAT^+^ B cells to the aorta plaques (46). Perhaps of relevance, therefore, in addition to suppressing glomerulonephritis in a Breg-dependent manner in the MRL/lpr mouse model of SLE, ES-62 strikingly reduces atherosclerotic lesions and the accompanying macrophage recruitment and fibrosis in the gld.apoE^(-/-)^ mouse model of lupus-accelerated atherosclerosis (56).

Finally, given the increasing evidence that non-neuronal sources of ACh suppress gut inflammation and promote intestinal barrier integrity (57), it is tempting to speculate that by resetting the BM myeloid/lymphoid bias to protect lymphopoiesis, ES-62 may harness ACh-producing B (and T) cells (46) to resolve inflammation and promote tissue repair resulting from disruption of the gut BM axis. Of relevance, it is well established that neuronal-derived ACh suppresses production of inflammatory cytokines, stimulates mucus production by goblet cells and acts on cryptic basal and stem cells to effect gut barrier repair (57). Indeed, ACh confers resistance to IL-1β-mediated gut pathology, as exemplified by disruption of tight junctions and loss of colon fucosylation and intestinal barrier integrity (28, 58). However, there is also increasing evidence that ACh release by ChAT-expressing haematopoietic and stromal cells plays roles in fighting bacterial infection and promoting epithelial barrier formation in the gut (57). Thus, ES-62 could potentially counteract the CIA-induced microbiome dysbiosis and associated loss of colon fucosylation and accompanying increase in IL-1β production and consequent cellular infiltration and gut pathology by promoting influx of ChAT-expressing ACh-secreting lymphoid cells.

## Supplementary Material

Supplementary Information

## Data Availability

The original contributions presented in the study are included in the article/Supplementary Material. Further inquiries can be directed to the corresponding authors.
