# Supplementary material for: The parasitic worm product ES-62 protects against collagen-induced arthritis by resetting the gut-bone marrow axis in a microbiome-dependent manner
Source: Front Trop Dis. Author manuscript; Available in PMC 2024 Mar 18. (PMC7615750; doi:10.3389/fitd.2023.1334705)

Supplementary Figures

Supplementary Figure 1. Representative gating strategies. For all cell populations **(A-C)**, BM cells were selected and dead cells and debris excluded on the basis of their forward versus side scatter **(i)** and singlet cells gated **(ii)**. **(A)** Identification of LSK HSCs by selection of Lin^- populations using a lineage-specific antibody cocktail and dump channel **(iii)** and gating of LSK HSCs as $Sca\text{-}1^+cKit^+$ ($CD117^+$) cells **(iv)**. **(B)** Identification of $RANKL^+$ BM populations by first gating on $CD45^-$ and $CD45^+$ populations **(iii),** the latter allowing for identification of $CD3^+$ (T cells) and $B220^+$ (B cells) **(iv)** and their respective $RANKL^+$ subpopulations **(v, vii)** and FMO controls **(vi, viii)**. The non-T, non-B $CD45^+$ $RANKL^+$ **(ix)**, $CD45^+RANKL^+$ **(xi)** and $CD45^-RANKL^+$ **(xiii)** cells and their FMO controls **(x, xii, xiv)** are shown. **(C)** For phenotyping of myeloid cells and OCPs, $CD3^-B220^-Ter119^-$ cells **(iii;** FMO **iv)** were gated to distinguish monocytes ($Ly6C^+Ly6G^-$) from neutrophils ($Ly6G^+$) (**v** and associated FMOs, **vi, vii),** with subsequent identification of $CD11b^{low}Ly6C^{high}Ly6G^-$ OCPs **(viii** and FMO, **ix)**.

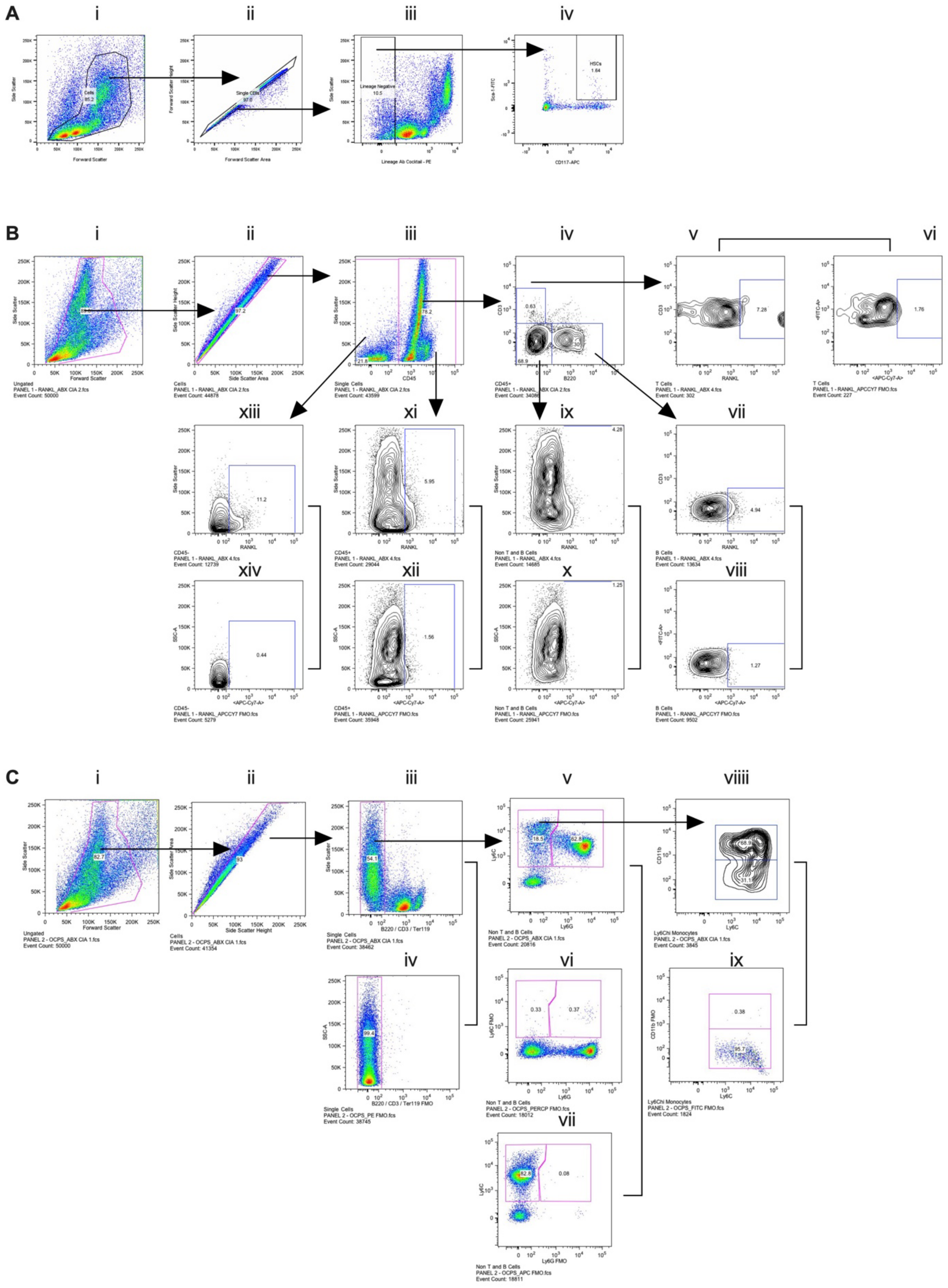

A
B
C

Supplementary Figure 2: Flow cytometric analysis of RANKL^{+} CD45^{-} and B220^{+} cells. Exemplar flow cytometric data underpinning the results presented in figures 5E and 5F. BM cells were selected and dead cells and debris excluded on the basis of forward versus side scatter **(i)** and doublet cells excluded **(ii)**. Gating on the CD45^{-} population **(iii)** allowed for the identification of CD45^{-}RANKL^{+} cells in the BM of a CIA-PBS mouse represented here as a histogram overlaid with the FMO control **(iv)**. In addition, a histogram is shown with overlays demonstrating the relative expression and MFI values (panel key) of CD45^{-} RANKL^{+} cells for each of a CIA-PBS and a CIA-ES-62 mouse and relevant FMO sample from Fig. 5E **(v)**. Likewise, gating on the CD45^{+} population for CD3^{+} and B220^{+} cells **(vi)** allowed for the identification of B220^{+}RANKL^{+} cells in the BM of the CIA-PBS mouse, (relative to FMO control) as shown by the histogram **(vii)**. In addition, a histogram is shown with overlays demonstrating the relative expression and MFI values (panel key) of B220^{+}RANKL^{+} cells for each of a CIA-PBS and a CIA-ES-62 mouse and a FMO sample from Fig. 5F **(viii)**.

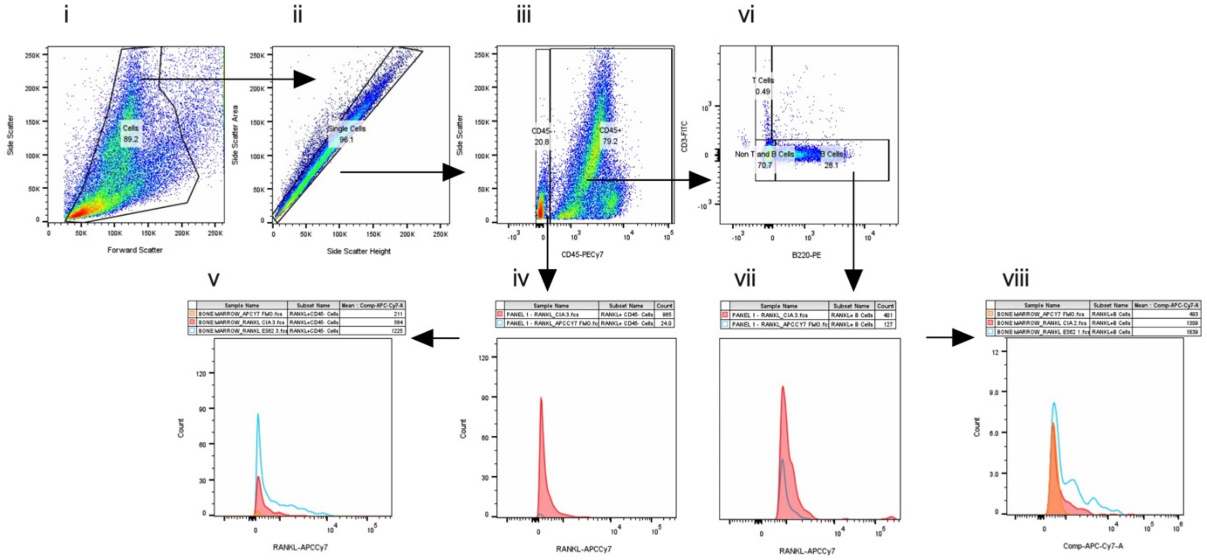

Supplement: Supplementary Information [file EMS194623-supplement-Supplementary_Information.pdf]
